# Child temperament and child-teacher relationship quality: Implications for children’s emotional functioning during preschool period

**DOI:** 10.3389/fpsyg.2022.992292

**Published:** 2022-11-10

**Authors:** Georgiana Susa-Erdogan, Oana Benga, Mihaela Albu-Răduleț, Teodora Macovei

**Affiliations:** Developmental Psychology Laboratory, Department of Psychology, Babeș-Bolyai University, Cluj-Napoca, Romania

**Keywords:** preschoolers, child temperament, internalizing and externalizing problems, child-teacher conflict, child-teacher closeness

## Abstract

Although, in the last years several studies have moved beyond analyzing the role of mother–child relationship in the association between child temperament and child emotional functioning, our knowledge is still limited about which fine-grained temperamental components of child reactivity and self-regulation are associated with child-teacher relationship quality. Also, fewer studies have looked at the moderating role of child-teacher relationship in the association between child temperament and child internalizing/externalizing problems during early childhood. The present study examined the relation between components of child temperamental Negative Affectivity, Surgency, and Effortful Control and child-teacher relationship quality (i.e., closeness, conflict) in preschool children. In addition, our aim was to test the moderating effect of the child-teacher relationship on the association between temperament and internalizing and externalizing problems. One hundred Romanian preschoolers (55 boys, mean age = 4.04 years) participated in this study. Mothers assessed their child’s temperament by completing the Children’s Behavior Questionnaire and externalizing and internalizing problems with the Child Behavior Checklist. Child-teacher relationship quality was evaluated by children’s teachers using the Student-Teacher Relationship Scale. Our results revealed that teachers rated their relationship as less conflictual with children who were assessed by their mothers as better in shifting and focusing attention, enjoying situations involving low stimulus intensity and displaying higher levels of Shyness, Sadness and Activity Level. Moreover, higher levels of Discomfort were associated with more conflict and less closeness while emotional reactivity such as Sadness, Fearfulness, and Activity Level were positively associated with closeness. Teacher-child closeness was associated with three temperamental self-regulation factors in the expected direction, except inhibitory control. Furthermore, results revealed a statistically significant interaction between child temperamental Shyness and child-teacher closeness in the prediction of child internalizing problems. Thus, when child-teacher closeness was low, there was a significant and positive relationship between child temperamental Shyness and child internalizing problems. Results highlight the importance of child-teacher relationship quality in relation to child temperament and social–emotional development during preschool period.

## Introduction

The perception teachers have of their relationship with their students plays an important role for children’s emotional and academic functioning (Sabol and Pianta, 2012; McCormick and O'Connor, 2015; Horn et al., 2021; Li et al., 2022). More specifically, teachers’ perception regarding relationship quality with their students (e.g., the degree of closeness and conflict) can affect the nature of child-teacher interaction within the classroom (e.g., engagement, communication) which can further impact children’s emotional experiences. An extensive body of research supports this view, many studies demonstrating that children who have a relationship with their teacher characterized by high levels of closeness (conceptualized as open, warmth and secure) experience fewer emotional and behavioral problems (Zatto and Hoglund, 2019; Bulotsky-Shearer et al., 2020; Harvey et al., 2022). Given the impact of teacher-student relationship quality on child emotional and behavioral functioning, it is critical to identify the factors related to higher levels of closeness and less conflict in the relationship between children and their teachers. Individual differences in child behavior in the form of temperamental characteristics is an important child-related factor that may influence teacher closeness and conflict. Child temperament, defined as individual differences that are observed in reactivity and self-regulation manifested on emotional, attentional and motor levels (Rothbart and Bates, 2006), is considered to influence the quality of child-teacher relationship. Rothbart’s multidimensional model of temperament (Putnam and Rothbart, 2006; Rothbart et al., 2007) considers that temperament encompasses two broad dimensions: reactivity and self-regulation. Reactivity includes Negative Affectivity which refers to individual differences in the propensity to experience and express negative emotions (i.e., Sadness, Fear, Anger/Frustration), as well as Surgency which is associated with expressions of positive emotions, high activity level, and reward seeking. Self-regulation, or Effortful Control, encompasses active and voluntary recruitment of higher-order cognitive processes, such as inhibitory control, high perceptual sensitivity, and attentional mechanisms that modulate reactivity.

When addressing temperamental reactivity, the large body of previous studies looked at Shyness as part of Surgency/Extraversion factor (Justice et al., 2008; Rudasill and Rimm-Kaufman, 2009; Arbeau et al., 2010; Bassett et al., 2017; Sette et al., 2019; Chen et al., 2021). Several cross-sectional studies found that children who displayed higher levels of Shyness have lowered teacher ratings of closeness (Justice et al., 2008; Sette et al., 2019) while longitudinal data showed that higher levels of child Shyness lead to less closeness in child-teacher relation over time (Arbeau et al., 2010; Rudasill, 2011). Moreover, in a recent study, Chen et al. (2021) analyzed the cultural differences between samples of Dutch and Chinese school-aged children regarding the role of children Shyness on child-teacher relationship. In this study, children reported on their own levels of Shyness and both teachers and children reported on the quality of their relationship. Results demonstrated that Shyness was associated with less closeness and more conflict as reported by children in both countries, but these associations were higher for the Chinese sample. Regarding teachers’ perception, only closeness was associated in both countries with children Shyness, namely higher Shyness was associated with less teacher-reported closeness. One possible explanation for this pattern of results regarding child Shyness and child-teacher closeness comes from ecological systems (Bronfenbrenner and Morris, 2007) and socialization theories (Grusec and Davidov, 2010), according to which children’s behaviors can shape the way caregivers, including teachers, respond and interact with them. As such, temperamental shy children who are inclined to show fearfulness and inhibited approach in the face of other people or novel situations (Rothbart and Bates, 2006) seek less comfort and support from their teachers and engage less positively with them. Therefore, teachers may subsequently perceive less closeness with these children. Since most cultures place value on autonomy, self-reliance, and sociability nowadays, due to globalization, shyness may often be regarded as a non-desirable trait and is discouraged by adults (Hofstede et al., 2010). Thus, shy children are more likely to be perceived negatively by teachers and hence may have fewer quality relations with them compared to non-shy children (Chen, 2019). Although, previous studies did not measure how teachers interpret children shyness, the study conducted by Rudasill and Rimm-Kaufman (2009) showed that Shyness was negatively related to child-teacher closeness indirectly through less frequent child-initiated interactions between teachers and children.

Other possible explanations for the relationship between Shyness and child-teacher relations can be grounded in the transactional model of development (Sameroff, 1975; Sameroff and Chandler, 1975) and temperamental theories (Rothbart, 2011). The transactional model of development considers that child Shyness and child-teacher relations can mutually influence each other. This theoretical perspective postulates that biologically based traits like temperament can be shaped by social context. While this hypothesis has not been studied compared to child-driven models, the few longitudinal child-teacher data reported in the literature are in line with the child-driven model, given that child Shyness predicted child-teacher closeness (Arbeau et al., 2010; Rudasill, 2011).

Fewer studies (e.g., Eisenberg et al., 2010; Diaz et al., 2017; Hernández et al., 2017; White et al., 2021) have investigated the impact of self-regulatory dimensions of temperament on child-teacher relationship. As such, Effortful Control has often been associated with more closeness and less conflict in the child-teacher relation (Eisenberg et al., 2010; Acar et al., 2021). Longitudinal evidence demonstrated that higher levels of Effortful Control, measured in kindergarten, positively predicted teacher-student closeness and strongly, negatively predicted teacher-student conflict 1 year later (Hernández et al., 2017). Other studies obtained the same results when also controlling for different child variables, such as SES and verbal intelligence (Valiente et al., 2012) or gender (Rudasill and Rimm-Kaufman, 2009). These results were interpreted mostly in terms of child-driven effects, namely that children with better self-regulatory abilities are easier to manage and they are able to interact more positively with others, which may contribute to higher quality relationship with their teachers (Nurmi, 2012).

From an attachment theory perspective, (Sroufe et al., 1999) higher quality of child-teacher relationship (warm and close) may enhance the development of self-regulation by supporting children to feel secure. In line with attachment theory, a recent study by Acar et al. (2021) showed that child-teacher closeness was positively associated with children’s self-regulation, whereas child-teacher conflict was negatively associated with children’s self-regulation. Moreover, the longitudinal study conducted by Goble et al. (2019) demonstrated that teachers’ emotional support across 1 year predicted gains in children’s inhibitory control development. In addition, teacher’s level of initial support in the beginning of the academic year moderated the relation between improvements in teachers’ emotional support and development of inhibitory control in children.

The research mentioned above concerning the relation between child temperament and child-teacher relationship has two major shortcomings. First, they have considered few temperamental traits with the majority of studies (for an exception, see Acar et al. (2021); White et al., 2021) looking at Shyness and Effortful Control. Looking at Shyness is reasonable given that this temperamental trait can place young children at risk for concurrent and long-term social- emotional challenges. However, there are other important dimensions of temperamental reactivity (e.g., Anger, Sadness, Activity Level) that are conceptually and empirically different from Shyness (Gartstein et al., 2012), which can impact child-teacher relationship. Equally, temperamental Effortful Control as a broader factor encompasses several abilities that can modulate reactivity: the ability to focus and shift attention, the ability to inhibit dominant responses and perform subdominant actions, the capacity to enjoy situations involving low stimulus intensity and to manifest positive emotions such as smiling or laughter. Because some individual differences in these fine-grained components of Effortful Control can have greater impact on the child-teacher relation (e.g., how much a child is able to enjoy activities with low stimulus intensity and flexibly shift attention when needed might be more applicable for learning contexts), analyzing the impact of these components of Effortful Control becomes relevant. Second, majority of this past research has been conducted with school-aged children and few studies addressed the preschool period (Zatto and Hoglund, 2019; Bulotsky-Shearer et al., 2020; Horn et al., 2021). However, preschool period is an important developmental stage for the experience of emotional difficulties, such as internalizing and externalizing problems. Preschool teachers represent key relational figures for children’s emotional and behavioral adjustment given that they interact with children several hours per day. Moreover, especially during early development, children’s temperamental dispositions may interact with features of environment, including the teacher relation context, to further predict children’s emotional functioning (Rothbart and Bates, 2006).

According to temperament theories (Rothbart and Bates, 2006) the consequences of child-teacher interaction on children’s emotional functioning may depend on children’s individual differences in temperamental reactivity and self-regulation. As such, the same type of interaction will be processed differently by children with different temperamental profiles. For instance, a shy child might be more likely to be affected by a less warm and close relation with their teachers while a supportive child-teacher interaction may protect against the negative impact of Shyness on child emotional functioning (Bulotsky-Shearer et al., 2020). Thus, temperamental models (Rothbart and Bates, 2006; Chess and Thomas, 2013) emphasize the moderating role of the environment that may support or hamper children’s adjustment.

A majority of previous research has analyzed the moderating role of family and in particular of mother–child relationship in the association between child temperament and child emotional functioning, while few studies have looked at child-teacher relational factors (Arbeau et al., 2010; Roubinov et al., 2017; Bulotsky-Shearer et al., 2020; White et al., 2021; Harvey et al., 2022). Roubinov et al. (2017) demonstrated that preschool children characterized by an overcontrolled temperamental profile (higher levels of Negative Affectivity and Effortful Control) produced more cortisol when they were at kindergarten only if their teachers reported less emotional and motivational support. Moreover, a recent study conducted by Harvey et al. (2022) found that low levels of Surgency were associated with fewer internalizing problems only for children that had a close relationship with their teachers while the longitudinal data obtained by Arbeau et al. (2010) demonstrated that for school-aged children shyness was significantly associated with anxiety and school avoidance only for children that had a child-teacher relationship characterized by low levels of closeness. In conclusion, previous studies have supported the prediction that child-teacher relationship can moderate the association between child temperament and child emotional functioning. However, these studies measured few fine-grained components of child temperament that may confer risk for internalizing and externalizing problems.

## Present study

Going beyond previous research, the present study examined the relation between fine-grained components of child temperamental Negative Affectivity, Surgency, and Effortful Control and child-teacher relationship quality (i.e., closeness, conflict) in preschool children. Further, our aim was to test the moderating effect of the child-teacher relationship quality on the association between temperament and internalizing and externalizing problems.

In order to advance the knowledge in this domain, first we conceptualized temperament based on fine-grained components of the two broad temperamental factors, namely reactivity and self-regulation (Rothbart, 2011). Second, we focused on preschool period, given that is the context in which many children experience the formation of a primary relation (i.e., child-teacher relation) outside the family environment for the first time. Third, we used different informants for the measurement of child-teacher relationship and child temperament. Based on previous data (Hernández et al., 2017; Acar et al., 2021), ecological systems (Bronfenbrenner and Morris, 2007), and socialization theories (Grusec and Davidov, 2010), we hypothesized that (a) fine-grained components of child Effortful Control would be associated with higher levels of closeness and less conflict in child-teacher relationship, while fine-grained components of child Negative Affectivity would be associated with lower levels of closeness and higher conflict. Regarding fine-grained components of Surgency, we expect positive associations between Smiling/Laughter and child-teacher closeness, while between Shyness, Activity Level, Impulsivity, High Intensity Pleasure, and closeness negative associations are anticipated. Regarding child-teacher conflict, we expect Impulsivity and High Intensity Pleasure to be associated with higher levels of child-teacher conflict, while Shyness and Smiling are expected to be associated with lower levels of child-teacher conflict. Based on temperamental theories postulating that children’s temperamental dispositions may interact with features of environment, we hypothesized that child-teacher relationship quality would moderate the association between children’s temperament and internalizing/externalizing problems. Developmental models of psychopathology (Nigg, 2006; Pérez-Edgar, 2019) and robust past research (Putnam and Stifter, 2005; Gartstein et al., 2017; Buss et al., 2018; Lin et al., 2018) have been postulated and have demonstrated that temperamental traits may be differentially associated with internalizing and externalizing problems. For example, higher levels of fearfulness, sadness, and shyness have been delineated as critical temperamental risk factors for internalizing problems, while higher levels of impulsivity, activity level, and anger have been identified for externalizing problems. Based on these theoretical models and empirical evidence, we expect that child-teacher closeness would decrease the strength of the association between temperamental risk (i.e., Fearfulness, Sadness and Shyness) on internalizing problems and between temperamental risk (i.e., Impulsivity, Activity Level, and High Intensity Pleasure) on externalizing problems. In contrast, conflict with teachers would increase the risk for experiencing internalizing problems and externalizing problems for children with higher levels in these temperamental traits.

### Participants

A total of 100 children (average age = 4.04 years, SD = 0.088, range = 2–5, 55 boys and 45 girls) and their primary caregivers, all mothers (average age = 34.12 years; SD = 5.39, range 26–50 years), participated in this study. A community-based sample was involved, the participants having been recruited *via* flyers distributed in local kindergartens. Mothers came from a middle- and upper-class backgrounds and their education level was diverse (on a scale ranging from 1, primary education, to 11, graduate studies), with 56% having a bachelor’s degree, 27% of them having a high school diploma, and the rest completed middle school and post-secondary education.

### Procedure

The procedure of the study was reviewed and approved by the Ethics Committee of Babeș-Bolyai University. At the beginning of the school year, parents were given informed consent forms; those who returned them were further offered the Children’s Behavior Questionnaire (CBQ) and the questionnaire pertaining to socio-demographic information. Parents filled in the questionnaires at home. In the middle of the school year (after 5–6 months), the primary teachers (those the child spent the most time with) also completed the Student-Teacher Relationship Scale (STRS), whereas the mothers additionally completed the Child Behavior Checklist (CBCL).

### Instruments

#### The children’s behavior questionnaire, standard form (CBQ)

For measuring child temperamental reactivity and emotion regulation, we used The Children’s Behavior Questionnaire, Standard Form (CBQ; Rothbart et al., 1994; Benga, 2004). The CBQ is a caregiver-report measure that assesses three major temperamental dimensions: Negative Affectivity, Effortful Control, and Surgency/Extraversion. The CBQ was developed for 3–7-year-old children and it features a total of 195 items and 15 subscales. Responses to the items are rated on a 7-point Likert scale, ranging from “extremely untrue of your child” (1) to “extremely true of your child”; additionally, the CBQ provides a “not applicable” alternative for items that do not apply to the child in question.

In regards to the current study, we used the child Negative Affectivity, Effortful Control, and Surgency dimensions. Negative Affectivity includes the following scales: Discomfort, Sadness, Fear, Anger/Frustration, and Soothability. Effortful Control contains Attentional Focusing, Inhibitory Control, Low Intensity Pleasure, and Perceptual Sensitivity. Surgency is defined by the following scales: Impulsivity, High Intensity Pleasure, Activity Level, and Shyness. Positive Anticipation and Smiling/Laughter also contribute to this factor.

According to Rothbart et al. (2001), Discomfort refers to negative affectivity related to the sensorial qualities of a stimulus, including intensity, rate, complexity, etc. (e.g., ‘Is not very bothered by pain’), Sadness refers to “negative affectivity and lowered mood and energy” due to the “exposure to suffering, disappointment, and object loss” (e.g., ‘Cries sadly when a favorite toy gets lost or broken’), and Fear covers “negative affectivity, including unease, worry, or nervousness, which is related to anticipated pain” (e.g., ‘Is not afraid of large dogs and/or other animals’). Anger/Frustration covers “negative affectivity related to interruption of ongoing tasks or goal blocking” (e.g., ‘Has temper tantrums when s(he) does not get what s(he) wants’) and Soothability refers to “the rate of recovery from peak distress, excitement, or general arousal” (‘Has a hard time setting down for a nap’).

Regarding the Effortful Control dimension, Attentional Focusing refers to “the capacity to maintain attentional focus on task-related channels” (‘When picking up toys or other jobs, usually keeps at the task until it’s done’), Inhibitory Control measures “the capacity to plan and to suppress inappropriate approach responses under instructions or novel or uncertain situations” (e.g., ‘Can lower his/her voice when asked to do so’), and Low Intensity Pleasure refers to pleasure or enjoyment derived from “situations involving low stimulus intensity” (e.g., ‘Rarely enjoys just being talked to’). Perceptual Sensitivity covers “the detection of slight, low-intensity stimuli from the external environment” (e.g., ‘Notices the smoothness or roughness of objects s(he) touches’) and Smiling/ Laughter measures the positive emotions in regards to changes in the intensity of a stimulus (e.g., ‘Laughs a lot at jokes and silly happenings’).

Impulsivity stands for the speed with which a response is initiated (e.g., ‘Usually rushes into an activity without thinking about it’) and High Intensity Pleasure refers to pleasure or enjoyment derived from “situations involving high stimulus intensity” (e.g., ‘Likes going down high slides or other adventurous activities’). Activity Level refers to the level of “gross motor activity, including rate and extent of locomotion” (e.g., ‘Seems always in a big hurry to get from one place to another’) and Shyness stands for “slow or inhibited … speed of approach and discomfort” in social interactions (e.g., ‘Often prefers to watch rather than join other children playing’). Additionally, Positive Anticipation refers to “the amount of excitement for expected pleasurable activities” (e.g., ‘Gets so worked up before an exciting event that s(he) has trouble sitting still’).

The CBQ has appropriate psychometric properties, with test–retest reliability and internal consistency ranging from.56 to.86, as obtained from the Romanian population. For the current sample, we also obtained appropriate reliability for the Effortful Control dimension (*α* = 0.71), for the Surgency dimension (*α* = 0.75), and for the Negative Affectivity dimension (*α* = 0.70). Internal consistency for fine-grained components of child temperamental Negative Affectivity was acceptable for Fear (*α* = 0.74), Anger (*α* = 0.88), Sadness (*α* = 0.76) but relatively low for Discomfort (*α* = 0.66). For Surgency, except for the Impulsivity (*α* = 0.50) the other subscales had good reliability (Shyness *α* = 0.79; Smiling *α* = 0.75, Activity Level *α* = 0.71, Approach *α* = 0.86, and High Intensity Pleasure *α* = 0.75). Finally, for fine-grained components of Effortful Control dimension was good for Inhibitory Control (*α* = 0.83) and Low Intensity Pleasure (*α* = 0.70) but relatively low for Perceptual Sensitivity (*α* = 0.68), Attentional Focusing (*α* = 0.58) and Attentional Shifting (*α* = 0.60). Although the reliability for some reactive and regulatory temperament variable appears low in the current study, this is similar with previous studies using CBQ in different samples (Putnam and Rothbart, 2006; White et al., 2011; Acar et al., 2021).

#### Student-teacher relationship scale

For evaluating the child-teacher quality of interaction, we used The Student-Teacher Relationship Scale, Short Form (Pianta, 2001). The STRS is a self-report instrument that measures the teacher’s relationship with individual children in their classroom (Pianta, 2001). It contains 15 items that are rated on a 5-point scale, with responses ranging from 1 (“definitely does not apply”) to 5 (“definitely applies”). The STRS covers two main dimensions, closeness and conflict. According to Pianta (2001), closeness refers to the degree to which the child-teacher relationship is characterized by warmth and positive affect, according to the teacher (e.g., ‘I share an affectionate, warm relationship with this child’), and conflict evaluates the degree to which teachers view interactions with the child as negative or disagreeable (e.g., ‘This child and I always seem to be struggling with each other’).

The STRS features good psychometric properties, with internal consistency ranging from.86 to.89 (Pianta and Steinberg, 1992) and good predictive and concurrent validity. For example, the questionnaire shows correlations with present and future academic skills (Hamre and Pianta, 2001), behavioral adjustment, and peer relations (Birch and Ladd, 1998). In the current study, the closeness dimension rendered good reliability (*α* = 0.81), whereas the conflict dimension featured very good reliability (*α* = 0.94).

#### The child behavior checklist 1½–5 years (CBCL 1½–5 years)

For measuring externalizing and internalizing problems, we used the caregiver version of the CBCL 1½–5 years (Achenbach and Rescorla, 2000). For the present study we calculated the total score for Internalizing dimension which includes the following subscales: Emotionally Reactive (e.g., ‘Disturbed by any change in routine’), Anxious/Depressed (e.g., ‘Nervous, high-strung, or tense’), Somatic Complaints (e.g., ‘Nausea, feels sick’), and Withdrawn (e.g., ‘Avoids looking others in the eye’). The Externalizing problems were measured with Attention Problems (e.g., ‘Cannot concentrate, cannot pay attention for long’) and Aggressive Behavior subscales (e.g., ‘Destroys things belonging to his/her family or other children’). Items are scored on a 3-point Likert scale (0 = false; 1 = somewhat true or sometimes true; 2 = very true or often true). The CBCL features good psychometric properties. The CBCL has shown good psychometric properties on the Romanian population as well, with high levels of test-rest reliability (Dobrean, 2009; Ştefan and Miclea, 2017) and internal consistency ranging from.85 to.94 (Ivanova et al., 2007a,b; Ştefan and Miclea, 2017). For the current study we obtained a very good reliability for the Internalizing (*α* = 0.91) and Externalizing (*α* = 0.94) dimensions.

## Results

Descriptive statistics for the study variables are presented in Table 1. Moreover, bivariate correlations between fine-grained components of child temperamental Negative Affectivity, Surgency, Effortful control, and child-teacher relationship are presented in Tables 2–4. The data yielded several statistically significant correlations of importance. Significant negative correlations were obtained between child-teacher conflict and fine-grained components of Effortful Control, namely, Low Intensity Pleasure, Inhibitory Control and a marginally significant negative correlation with Attentional Shifting, (*r* (98) = −0.19, *p* = 0.061). In addition, child-teacher conflict was also significantly and negatively correlated with the fine-grained component of Surgency, namely Shyness. Moreover, a significant positive correlation was found between child-teacher closeness and Activity Level as part of Surgency.

**Table 1:** Descriptive statistics for study variables.

Variable	*M*	*SD*	Range
*Temperament (CBQ)*			
Negative affectivity	4.44	0.51	3.79–5.53
Effortful control	4.78	0.43	3.95–5.66
Surgency	4.43	0.28	3.67–5.23
Internalizing problems (CBCL)	1.34	0.97	0–3.39
Externalizing problems (CBCL)	0.94	0.70	0–2.26
*Child-teacher relationship (STRS)*			
Child-teacher conflict	1.50	0.80	1–3.86
Child-teacher closeness	4.38	0.52	2.75–5

CBQ, Children’s Behavior Questionnaire; CBCL, Child Behavior Checklist; STRS, Student-Teacher Relationship Scale.

**Table 2:** Correlations for study variables (negative affectivity).

Variable	*n*	1	2	3	4	5	6	7
1. Child_Teacher_Conflict	100	—						
2. Child_Teacher_Closeness	100	−0.64^**^	—					
3. Discomfort	100	0.02	−0.11	—				
4. Fear	100	0.06	0.12	0.55^**^	—			
5. Anger/frustration	100	0.13	−0.02	0.65^**^	0.39^**^	—		
6. Sadness	100	0.03	−0.02	0.78^**^	0.18	0.72^**^	—	
7. Soothability	100	−0.12	−0.01	−0.33^**^	0.09	−0.33^**^	−0.39^**^	—

** *p* < 0.01.

**Table 3:** Correlations for study variables (effortful control).

Variable	*n*	1	2	3	4	5	6	7
1. Child_Teacher_Conflict	100	—						
2. Child_Teacher_Closeness	100	−0.64^**^	—					
3. Attentional focusing	100	−0.11	0.01	—				
4. Inhibitory control	100	−0.19^*^	0.02	0.67^**^	—			
5. Low Intensity pleasure	100	−0.20^*^	−0.03	0.27^**^	0.38^**^	—		
6. Perceptual sensitivity	100	0.10	0.10	0.11	0.18	−0.20^*^	—	
7. Attentional shifting	100	−0.19	−0.07	0.30^**^	0.49^**^	−0.12	0.39^**^	—

* *p* < 0.05;

** *p* < 0.01.

**Table 4:** Correlations for study variables (surgency).

Variable	*n*	1	2	3	4	5	6	7	8
1. Child_Teacher_Conflict	100	—							
2. Child_Teacher_Closeness	100	−0.64^**^	—						
3. Activity Level	100	0.01	0.26^**^	—					
4. Impulsivity	100	0.10	−0.07	0.39^**^	—				
5. High Intensity Pleasure	100	−0.12	0.12	0.41^**^	0.51^**^	—			
6. Shyness	100	−0.22^*^	−0.01	−0.20	−0.46^**^	−0.57^**^	—		
7. Approach	100	0.08	0.08	0.56^**^	0.55^**^	0.37^**^	−0.02	—	
8. Smiling/ Laughter	100	0.12	0.14	0.59^**^	0.29^**^	−0.12	−0.10	0.53^**^	—

* *p* < 0.05;

** *p* < 0.01.

### Child temperament and child-teacher relationship

In order to examine the relation between components of child temperamental Negative Affectivity, Surgency, and Effortful Control and child-teacher relationship quality two hierarchical regression analyzes (one with child-teacher conflict and one with child-teacher closeness as an outcome variable) were conducted (Tables 5, 6). The model predicting child-teacher conflict (Table 5) revealed statistical significance, *F* (5, 85) = 5, 27, *p* = 0.000 and explained 27% of the variance of conflict. Regarding fine-grained components of the Negative Affectivity, Discomfort was statistically significant and positively associated with conflict (*β* = 1.14, *p* = 0.008) while Sadness was statistically significant and negatively associated with conflict (*β* = −0.84, *p* = 0.010). Shyness and Activity Level, as part of Surgency, were negatively associated with conflict (*β* = −0.25, *p* = 0.019, *β* = −0.99 *p* = 0.005, respectively) and Smiling was positively associated with conflict (*β* = 1.03, *p* = 0.003). Low Intensity Pleasure, Attentional Shifting and Attentional Focusing were statistically significant and negatively associated with conflict (*β* = −0.30, *p* = 0.010, *β* = −0.24, *p* = 0.044, *β* = −0.44, *p* = 0.017, respectively). The second hierarchical regression model with child-closeness as an outcome (Table 6) was statistically significant, *F* (5, 85) = 2, 81, *p* = 0.021, and explained 20% of the variance of closeness. From Negative Affectivity, Discomfort significantly and negatively predicted closeness (*β* = −0.1.59, *p* = 0.001) while Fearfulness and Sadness were positively associated with closeness (*β* = 0.65, *p* = 0.028, *β* = 1.49, *p* = 0.000). For Surgency factor, only Activity Level predicted closeness (*β* = 0.73, *p* = 0.044). Attentional Focusing and Low Intensity Pleasure as part of Effortful Control were positively associated with closeness (*β* = 0.37, *p* = 0.054, *β* = 0.49, *p* = 0.046). In contrast, Inhibitory Control was negatively associated with closeness (*β* = −0.86, *p* = 0.045).

**Table 5:** Hierarchical regression coefficients for child temperament and child-teacher conflict.

Variable	Model 1			*t-*value	*p*-value
	*B*	*SE*	*β*		
Constant	0.85	0.37		2.28	0.025
Discomfort	1.18	0.43	1.14	2.71	0.008
Fear	−0.30	0.22	−0.37	−1.33	0.188
Anger	−0.00	0.19	−0.00	−0.00	0.997
Sadness	−1.01	0.38	−0.84	−2.62	0.010
Shyness	−0.28	0.12	−0.25	−2.39	0.019
Smiling	1.81	0.59	1.03	3.08	0.003
High intensity pleasure	0.40	0.30	0.37	1.35	0.179
Impulsivity	0.38	0.36	0.21	1.04	0.302
Activity level	−0.60	0.55	−0.99	−2.91	0.005
Inhibitory control	0.12	0.14	0.11	0.83	0.406
Low intensity pleasure	−0.40	0.15	−0.30	−2.62	0.010
Attentional shifting	−0.37	0.18	−0.24	−2.04	0.044
Attentional focusing	−0.57	0.23	−0.44	−2.43	0.017
Perceptual sensitivity	0.02	0.28	0.01	0.07	0.942

SE, standard error; Dependent variable: Child_Teacher_Conflict.

**Table 6:** Hierarchical regression coefficients for child temperament and child-teacher closeness.

Variable	Model 1			*t*-value	*p*-value
	*B*	*SE*	*β*		
Constant	−0.70	1.66		−0.42	0.675
Discomfort	−1.07	0.29	−1.59	−3.60	0.001
Fear	0.34	0.15	0.65	2.23	0.028
Anger	−0.25	0.13	−0.41	−1.86	0.065
Sadness	1.17	0.26	1.49	4.47	0.000
Shyness	0.27	0.14	0.37	1.87	0.064
Smiling	−0.36	0.40	−0.31	−0.88	0.377
High intensity pleasure	−0.13	0.20	−0.18	−0.65	0.517
Impulsivity	−0.01	0.24	−0.01	−0.05	0.960
Activity level	0.77	0.37	0.73	2.04	0.044
Inhibitory control	−0.58	0.29	−0.86	−2.03	0.045
Low intensity pleasure	0.42	0.20	0.49	2.02	0.046
Attentional shifting	0.29	0.24	0.29	1.21	0.227
Attentional focusing	0.31	0.16	0.37	1.95	0.054
Perceptual sensitivity	0.13	0.19	0.13	0.65	0.517

SE, standard error; Dependent variable: Child_Teacher_Closeness.

### The moderating role of child-teacher relationship on child temperament and internalizing/externalizing problems

Several regression analyzes were carried out using PROCESS for SPSS software (Preacher and Hayes, 2008) in order to analyze the moderating role of child-teacher relationship on child temperament and child internalizing and externalizing problems. In order to test our hypothesis that child-teacher closeness and conflict would moderate the association between children’s Impulsivity, Activity Level, High Intensity Pleasure and externalizing problems we run separate models to test these interaction effects. No interaction model was significantly associated with children’s externalizing problems (*b* = −0.02, *p* = 0.91 for Impulsivity x closeness, *b* = −0.03, *p* = 0.92 for Impulsivity x conflict, *b* = 0.12, *p* = 0.74 for Activity Level x closeness, *b* = −0.09, *p* = 0.66 for Activity Level x conflict, *b* = 0.19, *p* = 0.34 for High Intensity Pleasure x closeness, and *b* = −0.03, *p* = 0.77 for High Intensity Pleasure x conflict).

To test our hypothesis that child-teacher closeness and conflict would moderate the association between children’s Fearfulness, Shyness, Sadness and internalizing problems we also run separate models to test these interaction effects (Table 7). Only the model with children’s Shyness and child-teacher closeness as an interaction term significantly moderated the association between temperamental risk and internalizing problems (*b* = −0.73, *p* = 0.024).

**Table 7:** Hierarchical regression analysis for variables predicting child internalizing problems.

Variable	Estimate	*SE*	95% CI for B		*t*-value	*p*-value
			*LL*	*UL*		
CBQ_Shyness	0.22	0.14	−0.07	0.49	1.51	0.133
Child_Teacher_Closeness	−0.08	0.25	−0.58	0.43	−0.30	0.763
Child_Teacher_Conflict	−0.06	0.16	−0.39	0.26	−0.41	0.682
CBQ_Shyness × Child_Teacher_Closeness	−0.73	0.32	−1.36	−0.09	−2.28	0.024

SE, standard error; CI, confidence interval; LL, lower limit; UL, upper limit.

**Figure 1:**
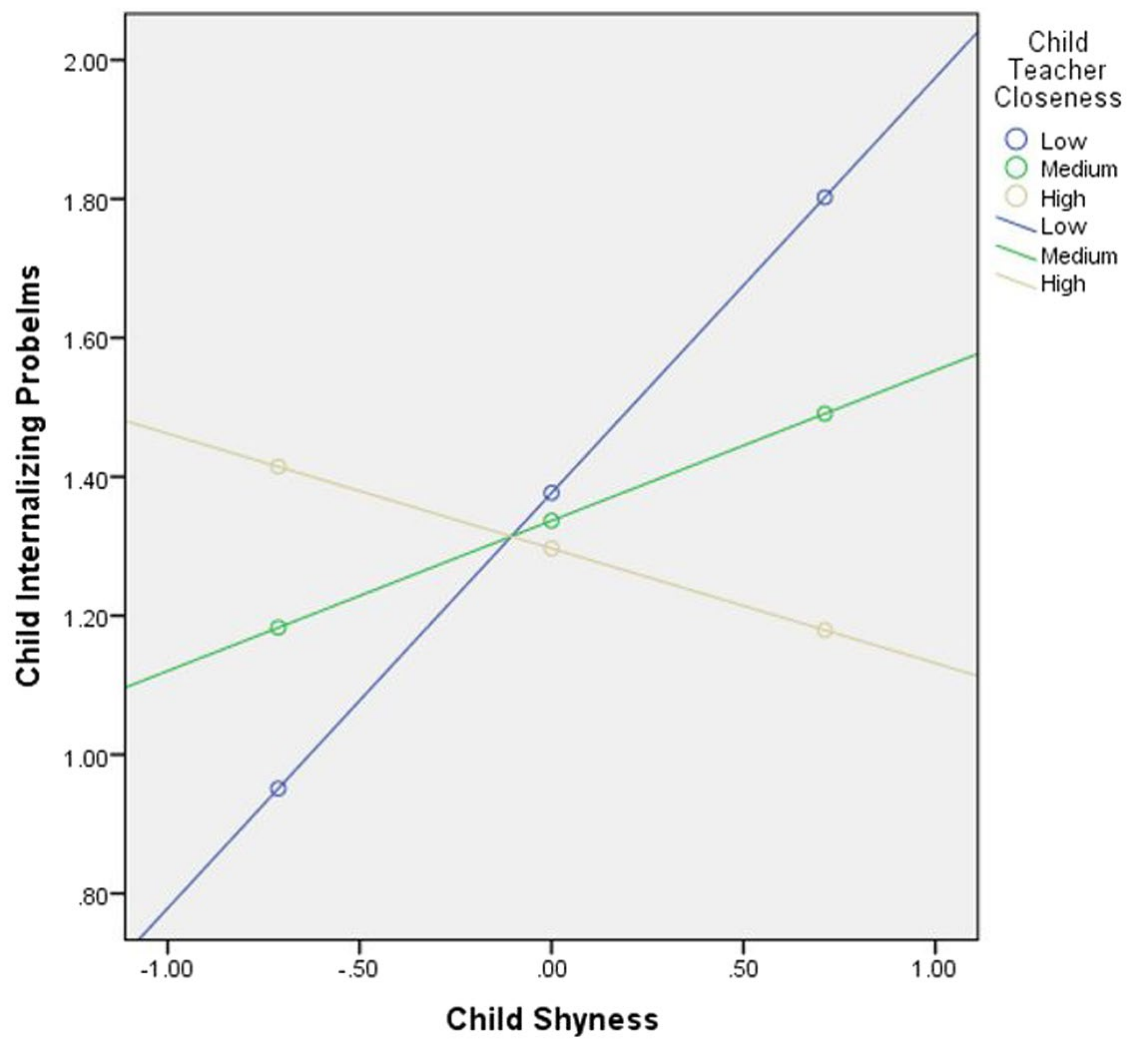
Interaction between child shyness and child-teacher closeness in predicting child internalizing problems.

As can be seen in Figure 1, the effect of child-teacher closeness was probed at low (−1 SD from mean), average, and high (+1 SD from mean) levels of child Shyness. When child-teacher closeness was low a significant and positive relationship between child Shyness and child internalizing problems was found (*b* = 0.60, *SE* = 0.23, *t* = 2.55, *p* = 0.012). However, when child-teacher closeness was high and medium, no statistically significant effect was found (*b* = −0.16, *SE* = 0.20, *p* = 0.42, *b* = 0.22, *SE* = 0.14, *p* = 0.13, respectively).

## Discussion

The present study aimed to examine child temperamental factors that are related to higher levels of closeness and less conflict in the relationship between children and their teachers during preschool period. In addition, we tested the moderating role of child-teacher interaction quality in the association between child temperamental risk and child internalizing/externalizing problems.

The results of the hierarchical regression analysis revealed that, three fine-grained components of child temperamental Effortful Control were significantly associated with child-teacher conflict. Specifically, children’s Low Intensity Pleasure, Attentional Shifting, and Attentional Focusing were associated with lower levels of conflict in the child-teacher relation. In addition, Shyness and Activity Level as reactive temperamental factors of Surgency dimension were associated with lower levels of child-teacher conflict, while Smiling as also part of Surgency was associated with higher levels of conflict. With respect to fine-grained components of negative affectivity we found that Discomfort was associated with higher conflict and Sadness with lower conflict. Child-teacher closeness was inversely associated with Discomfort and positively associated with Sadness, Fearfulness, and Activity Level. In addition, teacher-child closeness was associated with three temperamental self-regulation factors in the expected direction, except inhibitory control. That is, inhibitory control was inversely associated with closeness while Attentional Focusing and Low Intensity Pleasure were positively associated.

Another goal of the present study was to investigate the interactive role of child temperament and child-teacher relationship quality on child internalizing and externalizing problems. Regarding this goal, our study demonstrated a significant interaction effect between child Shyness and child-teacher closeness. Specifically, temperamental Shyness predicted higher levels of internalizing problems only when child-teacher closeness was low. However, temperamental Shyness did not predict internalizing problems when child-teacher closeness was high or medium.

Knowledge is limited about which fine-grained components of child temperamental reactivity and self-regulation explain more from the variance in child-teacher relationship during preschool. Our study addresses this shortcoming by showing that teachers rated their relationship as less conflictual with children who were assessed by their mothers as better in shifting and focusing attention, and enjoying situations involving low stimulus intensity. This result is in line with our hypothesis and confirms previous data demonstrating that higher temperamental self-regulation in children is linked with less conflict and more closeness in the child-teacher relation (Valiente et al., 2012; Hernández et al., 2017; White et al., 2021). Thus, young children’s attentional abilities, as well as their capacity to enjoy situations that are not highly arousing, such as being talked to, are critical for the establishment of a close and supportive relationship with their teachers. The reason these self-regulatory abilities are important for child-teacher relationship stems from the relevance these traits have in the learning context of the kindergarten. Specifically, much of the learning activities in kindergarten are teacher directed and conducted in groups in Romanian kindergartens. Therefore, children’s ability to shift attention toward teachers when needed, to inhibit inappropriate responses in order to follow teachers’ directions, and to be able to focus on tasks that involve low stimulus intensity, such as staying at a table and draw something, are traits that are highly appreciated and valued by teachers given their impact on the kindergarten learning environment. Contrary to our expectation we did not find a direct association between Shyness and child-teacher closeness but we found that teachers reported less conflict with children that were perceived by their mothers as having higher levels of Shyness. With respect to child-teacher conflict previous findings were inconsistent. For example, the meta-analysis conducted by Nurmi (2012) found that children shyness was not significantly associated with conflict. However, some studies found a positive association between child Shyness and conflict (Rudasill and Rimm-Kaufman, 2009; Sette et al., 2014; Chen et al., 2021) while others found a negative association (Rudasill, 2011). Thus, our results show that shy children were less likely to have conflictual relationships with teachers. This effect might be due to the fact that these children are initiating less interactions with their teachers. However, having less conflictual relationship with teachers may help preschool shy children to adapt to kindergarten environment. Not in line with our expectation we found that children who have higher levels of positive affect (Smiling) in response to changes in stimulus intensity, rate, complexity, and incongruity were more likely to be perceived by their teachers as being conflictual. One explanation for this result could be that teachers may perceive these children behavior as disturbing the learning environment given that they may act more exuberant during lessons. Moreover, although, our expectation was that higher Negative Reactivity would be associated with more child-teacher conflict our results demonstrate a more nuanced picture, depending on the type of negative emotional reactivity. That is, children with higher negative affect related to sensory stimulation (Discomfort) have less positive relationship with their teachers (i.e., higher conflict and lower closeness) while children with higher fearfulness have higher closeness and those with higher sadness have both less conflict and more closeness. This results can be interpret in the light of social functionalist theory of emotions (Campos et al., 1994; Keltner et al., 2022) which considers that each emotion motivates the behavior of others in a different way. For example, sadness and fear signal to others that support is needed, in contrast with discomfort that has a less clear message for the social environment. As such young children who are predispose to manifest fearfulness and sadness activates teacher’s comforting responses which in turn may increase child-teacher relationship quality.

Also, not in line with our expectation was the finding that teachers reported more closeness in relationships with children displaying higher levels of Activity Level as part of the Surgency dimension. In addition, children with higher inhibitory control had less closeness with their teachers. Regarding the relation between Activity Level and closeness it is possible that children who are active and move a lot from one place to another are more likely to also initiate more interactions with their teachers and to make more bids for attention from them, which may facilitate the formation of positive child-teacher relationships given that close relationships require frequent interactions. In line with this argument, the study conducted by Rudasill and Rimm-Kaufman (2009) showed that less shy children initiated more interactions with teachers compared to shy ones. Although the relation between inhibitory control and closeness detected in the present study needs to be replicated in future research, it is possible that young children who are better in controlling their behavior receive less attention and contact from their teachers.

Regarding the moderating role of child-teacher interaction quality in the association between child temperament and child internalizing/externalizing problems, our hypothesis was partially supported since only child-teacher closeness acted as a moderator of the relationship between Shyness and internalizing problems. Specifically, when teachers reported having less closeness, children presenting higher levels of Shyness experienced more internalizing problems. This result is similar with other studies that demonstrated the protective role of high levels of closeness in child-teacher relation for shy children that have difficulties approaching people and new situations (Arbeau et al., 2010; Harvey et al., 2022), and with temperamental theories that postulate an interaction between temperamental traits and context (Rothbart and Bates, 2006; Chess and Thomas, 2013). However, no moderating effect was observed between other temperamental components and closeness in the prediction of internalizing or externalizing problems. In addition, conflict was not identified as a moderator of the relationship between temperament and internalizing or externalizing problems. In our sample, teachers rated overall low levels of conflict in their relationship with children, which can explain this null result regarding conflict. Moreover, the finding that no direct or interactive effects were found between child temperament, child-teacher relation (both conflict and closeness), and externalizing problems can be explained by the low levels of externalizing problems reported by parents in this community-based sample. It might be that parents are less accurate in reporting children externalizing problems, as compared to internalizing problems, and future studies should also include teachers’ reports. Our results are in contrast with previous data reported by Harvey et al. (2022) in school-aged children, where a direct effect was found between temperament and externalizing problems but no moderation from child-teacher relationship quality. However, compared to our study, those children were presenting higher levels of externalizing problems at study entry (t-scores corresponding to the cutoff for high risk).

### Strengths and limitations

This study has several contributions for the research focused on identifying child factors associated with teacher-student relationship quality during early development. First, it looks at which fine-grained components of child temperamental reactivity and self-regulation have greater impact on child-teacher relationship during preschool. Second, we focused on the preschool period given that young children’s adaptation to the kindergarten environment is a challenging developmental task that can be facilitated by the relationship children form with their teachers. Third, we analyzed joint effects between child temperament and child-teacher interaction quality in predicting internalizing and externalizing problems in children. Limitations include: the lack of multiple measurement time-points for child temperament and child-teacher relationship quality, which did not allow us to test the direction of influences, as well as stability and change within child-teacher relationships as a function of child temperament; the lack of a direct observational measure of child-teacher interactions, given that teachers might report less conflict with children due to social desirability; the use of one respondent (i.e., the mother) for the assessment of internalizing and externalizing problems.

## Conclusion

The present study highlights the unique contribution of several fine-grained components of child temperament on the establishment and maintenance of a less conflictual and closer relationship with their teachers. In addition, our study underscores the importance of the quality of child-teacher relationship for shy children. This result is especially relevant for school psychologists or other mental health professional who would like to provide support for teachers in order to facilitate their ability to support children experiencing higher levels of shyness. These children may require extra support to engage in the kindergarten environment which can challenge teachers and as a consequence may hinder the formation of a positive relationships with them.

## Data availability statement

The raw data supporting the conclusions of this article will be made available by the authors, without undue reservation.

## Ethics statement

The studies involving human participants were reviewed and approved by Ethics Committee of Babeș-Bolyai University. Written informed consent to participate in this study was provided by the participants' legal guardian/next of kin.

## Author contributions

GS-E contributed to study design, processing, statistical analysis, interpretation, paper writing, and review. OB and TM contributed to paper writing and review. MA-R contributed to data collection. All authors contributed to the article and approved the submitted version.

## Conflict of interest

The authors declare that the research was conducted in the absence of any commercial or financial relationships that could be construed as a potential conflict of interest.

## Publisher’s note

All claims expressed in this article are solely those of the authors and do not necessarily represent those of their affiliated organizations, or those of the publisher, the editors and the reviewers. Any product that may be evaluated in this article, or claim that may be made by its manufacturer, is not guaranteed or endorsed by the publisher.

## References

[ref1] Acar I. H. Torquati J. C. Raikes H. Rudasill K. M. (2021). Pathways to low-income children’s self-regulation: child temperament and the qualities of teacher–child relationships. Early Educ. Dev. 32, 1103–1121. doi: 10.1080/10409289.2020.1830465

[ref2] Achenbach T. M. Rescorla L. A. (2000). Manual for the ASEBA Preschool Forms and Profiles. Burlington, VT: University of Vermont, Research Center for Children, Youth, & Families.

[ref4] Arbeau K. A. Coplan R. J. Weeks M. (2010). Shyness, teacher-child relationships, and socio-emotional adjustment in grade 1. Int. J. Behav. Dev. 34, 259–269. doi: 10.1177/0165025409350959

[ref5] Bassett H. H. Denham S. A. Fettig N. B. Curby T. W. Mohtasham M. Austin N. (2017). Temperament in the classroom: children low in surgency are more sensitive to teachers’ reactions to emotions. Int. J. Behav. Dev. 41, 4–14. doi: 10.1177/0165025416644077

[ref6] Benga O. (2004). Dezvoltarea cogniţiei sociale la copii Cluj-Napoca: Editura ASCR.

[ref7] Birch S. H. Ladd G. W. (1998). Children's interpersonal behaviors and the teacher–child relationship. Dev. Psych. 34, 934–946. doi: 10.1037/0012-1649.34.5.934 9779740

[ref9] Bronfenbrenner U. Morris P. A. (2007). “The bioecological model of human development,” in Handbook of Child Psychology. eds. Damon W. Lerner R. M. Lerner R. M. (Hoboken, NJ: Wiley), 793–828.

[ref10] Bulotsky-Shearer R. J. Fernandez V. A. Bichay-Awadalla K. Bailey J. Futterer J. Qi C. H. (2020). Teacher-child interaction quality moderates social risks associated with problem behavior in preschool classroom contexts. J. Appl. Dev. Psychol. 67:101103. doi: 10.1016/j.appdev.2019.101103

[ref11] Buss K. A. Davis E. L. Ram N. Coccia M. (2018). Dysregulated fear, social inhibition, and respiratory sinus arrhythmia: a replication and extension. Child Dev. 89, e214–e228. doi: 10.1111/cdev.12774, PMID: 28326533 PMC5608616

[ref12] Campos J. J. Mumme D. Kermoian R. Campos R. G. (1994). A functionalist perspective on the nature of emotion. Jap. J. Res. Emot. 2, 1–20. doi: 10.4092/jsre.2.1 7984165

[ref13] Chen X. (2019). Culture and shyness in childhood and adolescence. New Ideas Psych. 53, 58–66. doi: 10.1016/j.newideapsych.2018.04.007

[ref14] Chen M. Zee M. Roorda D. L. (2021). Students' shyness and affective teacher-student relationships in upper elementary schools: a cross-cultural comparison. Learn. Individ. Differ. 86:101979. doi: 10.1016/j.lindif.2021.101979

[ref15] Chess S. Thomas A. (2013). Goodness of Fit: Clinical Applications, From Infancy through Adult Life. New York: Routledge.

[ref16] Diaz A. Eisenberg N. Valiente C. Van Schyndel S. Spinrad T. L. Berger R.. (2017). Relations of positive and negative expressivity and effortful control to kindergarteners’ student–teacher relationship, academic engagement, and externalizing problems at school. J. Res. Pers. 67, 3–14. doi: 10.1016/j.jrp.2015.11.002, PMID: 28584388 PMC5455333

[ref17] Dobrean A. (2009). The Romanian Version of the Achenbach System of Empirically Based Assessment. Cluj-Napoca: RTS Publishing.

[ref18] Eisenberg N. Valiente C. Eggum N. D. (2010). Self-regulation and school readiness. Early Educ. Dev. 21, 681–698. doi: 10.1080/10409289.2010.497451, PMID: 21234283 PMC3018834

[ref19] Gartstein M. A. Prokasky A. Bell M. A. Calkins S. Bridgett D. J. Braungart-Rieker J.. (2017). Latent profile and cluster analysis of infant temperament: comparisons across person-centered approaches. Dev. Psych. 53, 1811–1825. doi: 10.1037/dev0000382, PMID: 28758787 PMC5612890

[ref20] Gartstein M. A. Putnam S. P. Rothbart M. K. (2012). Etiology of preschool behavior problems: contributions of temperament attributes in early childhood. Infant Ment. Health J. 33, 197–211. doi: 10.1002/imhj.21312, PMID: 28520102

[ref21] Goble P. Sandilos L. E. Pianta R. C. (2019). Gains in teacher-child interaction quality and children's school readiness skills: does it matter where teachers start? J. Sch. Psychol. 73, 101–113. doi: 10.1016/j.jsp.2019.03.006, PMID: 30961876

[ref22] Grusec J. E. Davidov M. (2010). Integrating different perspectives on socialization theory and research: a domain-specific approach. Child Dev. 81, 687–709. doi: 10.1111/j.1467-8624.2010.01426.x, PMID: 20573097

[ref23] Hamre B. K. Pianta R. C. (2001). Early teacher–child relationships and the trajectory of children's school outcomes through eighth grade. Child Dev. 72, 625–638. doi: 10.1111/1467-8624.00301, PMID: 11333089

[ref24] Harvey E. Lemelin J. P. Déry M. (2022). Student-teacher relationship quality moderates longitudinal associations between child temperament and behavior problems. J. Sch. Psychol. 91, 178–194. doi: 10.1016/j.jsp.2022.01.007, PMID: 35190075

[ref25] Hernández M. M. Valiente C. Eisenberg N. Berger R. H. Spinrad T. L. Van Schyndel S. K.. (2017). Elementary students’ effortful control and academic achievement: the mediating role of teacher–student relationship quality. Early Child Res. Q. 40, 98–109. doi: 10.1016/j.ecresq.2016.10.004, PMID: 28684888 PMC5495479

[ref26] Hofstede G. Hofstede G.J. Minkov M. (2010). Cultures and Organizations. 3rd Edn. New York: McGraw-Hill.

[ref27] Horn E. P. McCormick M. P. O’Connor E. E. McClowry S. G. Hogan F. C. (2021). Trajectories of teacher–child relationships across kindergarten and first grade: the influence of gender and disruptive behavior. Early Child Res. Q. 55, 107–118. doi: 10.1016/j.ecresq.2020.10.003

[ref28] Ivanova M. Y. Achenbach T. M. Dumenci L. Rescorla L. A. Almqvist F. Weintraub S.. (2007a). Testing the 8-syndrome structure of the child behavior checklist in 30 societies. J. Clin. Child Adolesc. Psychol. 36, 405–417. doi: 10.1037/0022-006X.75.5.729, PMID: 17658984

[ref29] Ivanova M. Y. Achenbach T. M. Rescorla L. A. Dumenci L. Almqvist F. Bathiche M.. (2007b). Testing the Teacher’s report form syndromes in 20 societies. School Psych. Rev. 36, 468–483. doi: 10.1080/02796015.2007.12087934

[ref30] Justice L. M. Cottone E. A. Mashburn A. Rimm-Kaufman S. E. (2008). Relationships between teachers and preschoolers who are at risk: contribution of children's language skills, temperamentally based attributes, and gender. Early Educ. Dev. 19, 600–621. doi: 10.1080/10409280802231021

[ref31] Keltner D. Sauter D. Tracy J. L. Wetchler E. Cowen A. S. (2022). How emotions, relationships, and culture constitute each other: advances in social functionalist theory. Cognit. Emot. 36, 388–401. doi: 10.1080/02699931.2022.2047009, PMID: 35639090

[ref32] Li L. Valiente C. Eisenberg N. Spinrad T. L. Johns S. K. Berger R. H.. (2022). Longitudinal associations among teacher–child relationship quality, behavioral engagement, and academic achievement. Early Child Res. Q. 61, 25–35. doi: 10.1016/j.ecresq.2022.05.006

[ref33] Lin B. Ostlund B. D. Conradt E. Lagasse L. L. Lester B. M. (2018). Testing the programming of temperament and psychopathology in two independent samples of children with prenatal substance exposure. Dev. Psychopathol. 30, 1023–1040. doi: 10.1017/S0954579418000391, PMID: 30068412 PMC6074047

[ref34] McCormick M. P. O'Connor E. E. (2015). Teacher–child relationship quality and academic achievement in elementary school: does gender matter? J. Educ. Psych. 107, 502–516. doi: 10.1037/a0037457

[ref35] Nigg J. T. (2006). Temperament and developmental psychopathology. J. Child Psychol. Psychiatry 47, 395–422. doi: 10.1111/j.1469-7610.2006.01612.x 16492265

[ref36] Nurmi J. E. (2012). Students’ characteristics and teacher–child relationships in instruction: a meta-analysis. Educ. Res. Rev. 7, 177–197. doi: 10.1016/j.edurev.2012.03.001

[ref37] Pérez-Edgar K. (2019). “Through the looking glass: temperament and emotion as separate and interwoven constructs,” in Handbook of Emotional Development. eds. LoBue V. Pérez-Edgar K. Buss K. (Cham, Switzerland: Springer), 139–168.

[ref38] Pianta R. C. (2001). Student-Teacher Relationship Scale: Professional Manual. Odessa, FL: Psychological Assessment Resources.

[ref39] Pianta R. C. Steinberg M. (1992). Teacher-child relationships and the process of adjusting to school. New Dir. Child Adolesc. Dev. 1992, 61–80. doi: 10.1002/cd.23219925706

[ref1001] Preacher K. J. Hayes A. F. (2008). Assessing mediation in communication research sourcebook of advanced data analysis methods for communication research. London: The Sage, 13–54.

[ref40] Putnam S. P. Rothbart M. K. (2006). Development of short and very short forms of the children’s behavior questionnaire. J. Pers. Assess. 87, 102–112. doi: 10.1207/s15327752jpa8701_09 16856791

[ref41] Putnam S. P. Stifter C. A. (2005). Behavioral approach–inhibition in toddlers: prediction from infancy, positive and negative affective components, and relations with behavior problems. Child Dev. 76, 212–226. doi: 10.1111/j.1467-8624.2005.00840.x, PMID: 15693768

[ref42] Rothbart M. K. (2011). Becoming Who We Are: Temperament and Personality in Development. New York: Guilford Press.

[ref43] Rothbart M. K. Ahadi S. A. Hershey K. L. (1994). Temperament and social behavior in childhood. Merrill-Palmer Q. 1982, 21–39.

[ref44] Rothbart M. K. Ahadi S. A. Hershey K. L. Fisher P. (2001). Investigations of temperament at three to seven years: the Children's behavior questionnaire. Child Dev. 72, 1394–1408. doi: 10.1111/1467-8624.00355, PMID: 11699677

[ref1006] Rothbart M. K. Sheese B. E. Posner M. I. (2007). Executive attention and effortful control: Linking temperament, brain networks, and genes. Child Dev. Perspect. 1, 2–7. doi: 10.1111/j.1750-8606.2007.00002.x

[ref45] Rothbart M. K. Bates J. E. (2006). “Temperament,” in Handbook of Child Psychology: Social, Emotional, and Personality Development. eds. Eisenberg N. Damon W. Lerner R. M. (New York: John Wiley & Sons, Inc.), 99–166.

[ref46] Roubinov D. S. Hagan M. J. Boyce W. T. Essex M. J. Bush N. R. (2017). Child temperament and teacher relationship interactively predict cortisol expression: the prism of classroom climate. Dev. Psychopathol. 29, 1763–1775. doi: 10.1017/S0954579417001389, PMID: 29162182 PMC9662253

[ref47] Rudasill K. M. (2011). Child temperament, teacher–child interactions, and teacher–child relationships: a longitudinal investigation from first to third grade. Early Child Res. Q. 26, 147–156. doi: 10.1016/j.ecresq.2010.07.002

[ref48] Rudasill K. M. Rimm-Kaufman S. E. (2009). Teacher–child relationship quality: the roles of child temperament and teacher–child interactions. Early Child Res. Q. 24, 107–120. doi: 10.1016/j.ecresq.2008.12.003

[ref49] Sabol T. J. Pianta R. C. (2012). Recent trends in research on teacher–child relationships. Attach Hum. Dev. 14, 213–231. doi: 10.1080/14616734.2012.672262, PMID: 22537521

[ref1002] Sameroff A. (1975). Transactional models in early social relations. Hum. Dev. 18, 65–79. doi: 10.1159/000271476

[ref1003] Sameroff A. J. Chandler M. J. (1975). “Reproductive risk and the continuum of caretaking casualty,” in Handbook of Child Psychology: Social, Emotional, and Personality Development. eds. Horowitz F. D. Hetherington E. M. Scarr-Salapatek S. Siegel G. M. (Chicago: University of Chicago Press), 187–244.

[ref50] Sette S. Baldwin D. Zava F. Baumgartner E. Coplan R. J. (2019). Shame on me? Shyness, social experiences at preschool, and young children’s self-conscious emotions. Early Child Res. Q. 47, 229–238. doi: 10.1016/j.ecresq.2018.12.012

[ref51] Sette S. Baumgartner E. Schneider B. H. (2014). Shyness, child–teacher relationships, and socio-emotional adjustment in a sample of Italian preschool-aged children. Infant Child Dev. 23, 323–332. doi: 10.1002/ICD.1859

[ref52] Sroufe L. A. Carlson E. A. Levy A. K. Egeland B. (1999). Implications of attachment theory for developmental psychopathology. Dev. Psychopathol. 11, 1–13. doi: 10.1017/S0954579499001923 10208353

[ref1004] Ştefan C. A. Miclea M. (2017). Reliability and Validity of Two Brief Screening Measures of Preschoolers’ Social–Emotional Competencies. Sch. Ment. Health 9, 44–65. doi: 10.1007/s12310-016-9200-5

[ref1005] Valiente C. Swanson E. Eisenberg N. (2012). Linking students’ emotions and academic achievement: When and why emotions matter. Child Dev. Perspect. 6, 129–135. doi: 10.1111/j.1750-8606.2011.00192.x 23115577 PMC3482624

[ref53] White L. K. McDermott J. M. Degnan K. A. Henderson H. A. Fox N. A. (2011). Behavioral inhibition and anxiety: the moderating roles of inhibitory control and attention shifting. J. Abnorm. Child Psychol. 39, 735–747. doi: 10.1007/s10802-011-9490-x, PMID: 21301953 PMC3624966

[ref54] White A. S. Sirota K. M. Frohn S. R. Swenson S. E. Rudasill K. M. (2021). Temperamental constellations and school readiness: a multivariate approach. Int. J. Environ. Res. Public Health 18:55. doi: 10.3390/ijerph18010055 PMC7795607 33374772

[ref1007] Zatto B. R. Hoglund W. L. (2019). Children’s internalizing problems and teacher–child relationship quality across preschool. Early Child Res. Q. 49, 28–39. doi: 10.1016/j.ecresq.2019.05.007

